# Executive Functions and Reading Skills in Low-Risk Preterm Children

**DOI:** 10.3390/children12081011

**Published:** 2025-07-31

**Authors:** Miguel Pérez-Pereira, Constantino Arce, Anastasiia Ogneva

**Affiliations:** 1 Institute of Psychology, University of Santiago de Compostela, 15782 Santiago de Compostela, Spain; constantino.arce@usc.es; 2 Department of Developmental and Educational Psychology, University of Santiago de Compostela, 15782 Santiago de Compostela, Spain; 3 Department of Social Psychology, Basic Psychology, and Methodology, University of Santiago de Compostela, 15782 Santiago de Compostela, Spain; 4 Center for Language, Brain and Learning, UiT–The Arctic University of Norway, 9019 Tromsø, Norway; anastasiia.ogneva@uit.no

**Keywords:** low-risk preterm children, reading skills, executive functions, longitudinal effects, predictive effects, prematurity

## Abstract

**Highlights:**

Executive functions (EFs) at ages 4, 5, and 8 and their relationship with reading abilities at age 9 were studied in 111 low-risk preterm children across three gestational age (GA) groups and 34 full-term children. No significant differences in EFs or reading abilities were found between GA groups. Linear regression analyses revealed that memory predicted lower-level reading processes (such as letter names or word reading), while higher-level reading processes (grammatical structures, sentence comprehension, and text comprehension) were affected by higher-order EFs, such as cognitive flexibility or planning.

These findings suggest that low-risk preterm children exhibit similar developmental trajectories in EFs and reading skills as full-term peers and highlight the role of EFs in supporting several components of reading.

**What are the main findings?**

- No significant difference was found between full-term and low-risk preterm children in their executive functions and their reading skills. Executive functions were assessed using cognitive and behavioral scale measures taken at different ages (4, 5, and 8 years), while reading skills were assessed at 9 years of age.
- Executive functions have a low-to-moderate predictive effect on reading skills. The effects of the different executive functions vary depending on the reading process. Verbal and non-verbal working memory had a positive significant effect on decoding skills. Cognitive flexibility and planning, as well as inhibitory control, showed positive effects on reading comprehension skills.

**What is the implication of the main finding?**

- There are differences between low-risk and high-risk preterm children in their competencies in reading and in executive functions. It is important to specify the type of population studied.
- Early assessment of executive functions is relevant given the long-lasting effects of executive functions on reading.

**Abstract:**

**Background/Objectives.** Previous research with extremely and very preterm children indicates that these children obtain significantly lower results in executive functions (EFs) and in reading skills than full-term (FT) children. The comparison results do not seem to be so clear when other PT children in lower-risk conditions are studied. Many studies with typically developing and preterm (PT) children indicate that reading ability is determined, in part, by EFs. Therefore, the study of EFs and reading and their relationships in low-risk PT children is pertinent. **Methods.** In the present study, 111 PT children, classified into three groups with different ranges of gestational age (GA), and one group of 34 FT children participated in a longitudinal study, carried out from 4 to 9 years of age. The results obtained from the four groups in different EFs measured at 4, 5, and 8 years of age, and in reading skills at 9 years of age were compared. The possible effects of EFs on reading skills were studied through multiple linear regression analyses. **Results.** The results obtained indicate that no significant difference was found between FT children and any of the GA groups of PT children, either in EFs or reading skills. The effect of EFs on reading skills was low to moderate. Verbal and non-verbal working memory had a positive significant effect on decoding skills (letter names, same–different, and word reading), but not on reading comprehension processes. Higher-order EFs (cognitive flexibility and planning), as well as inhibitory control, showed positive effects on reading comprehension skills. The effects of the different EFs varied depending on the reading process. **Conclusions.** In conclusion, low-risk PT children do not differ from FT children in their competence in EFs or reading skills. There are long-lasting effects of EFs, measured several years before, on reading skills measured at 9 years of age.

## 1. Introduction

The acquisition of literacy skills is a cognitively demanding process that entails the coordinated development of multiple skills, including visual perceptual abilities, language skills, working memory, attentional control, etc. [1]. Reading involves both lower-level processes, such as letter identification and word recognition, and higher-level operations that enable the construction of meaning from a text [2]. While decoding individual words is fundamental, effective reading comprehension requires the integration of words into syntactic structures and coherent discourse. Higher-level comprehension processes involve both bottom-up mechanisms, such as automatic word recognition and lexical activation. They also depend on top-down strategies, like contextual inference and predictive processing [3]. Although empirical evidence suggests that the efficiency of lower-level decoding significantly influences higher-order comprehension [4], the nature of the interplay between these processing levels remains insufficiently understood [5].

In addition to well-established predictors of reading ability, such as phonological awareness, rapid automatized naming, and oral language proficiency [6,7], a growing body of research highlights the role of executive functions (EFs) in literacy development. EFs are increasingly recognized as important to early reading acquisition and later reading comprehension [8,9,10,11,12,13,14]. Studies have shown that verbal inhibitory control is predictive of early reading skills [1] and that EFs contribute to comprehension, both directly and indirectly, through phonological awareness and vocabulary [15,16]. Moreover, some research suggests EFs can moderate the relationship between decoding and comprehension, in line with the simple view of reading [17]. For example, updating has been identified as a key predictor of reading comprehension among primary school students [18], while inhibitory control is crucial for filtering irrelevant information during reading [19,20].

Research also shows that EF deficits may align with specific literacy outcomes. Children with both reading and spelling difficulties tend to exhibit broader EF deficits (e.g., planning, attention, and working memory, among others), whereas those with isolated deficits may show more selective executive weaknesses [21]. Planning and working memory have demonstrated direct predictive effects on reading comprehension, and inhibitory control contributes indirectly via decoding [22,23]. A meta-analysis confirmed moderate positive correlations between EFs and reading, particularly comprehension, with the most robust associations for working memory and shifting [10]. However, this relationship is complex, as some studies suggest that once language skills are accounted for, the predictive value of EFs diminishes [24,25,26]. In some cases, EFs predict reading comprehension but not word recognition [14].

Studies in preterm (PT) children have shown that those born very (VPT) or extremely preterm (EPT) often exhibit small-to-moderate EF deficits, particularly in working memory, attention, and planning [27,28,29,30,31,32,33,34,35]. However, not all EF skills are equally affected, and findings from cognitive and behavioral measures are sometimes inconsistent [35,36]. It is also important to take into consideration that extremely and very preterm children constitute only around 20% of the total population of PT children, and that children of lower GA have higher risks of suffering biomedical complications, which have a detrimental effect on neurodevelopment [37]. For this reason, studies on EFs carried out with children of a wider GA range, and without additional biomedical complications, are necessary in order to obtain a more complete picture.

Recent studies, including children born moderate and late preterm (MLP), have found more subtle EF difficulties. While some evidence indicates differences in working memory between MLP and full-term (FT) children [38], other studies with low-risk PT samples report no significant differences in executive attention or inhibitory control [7,39]. A meta-analysis reported that MLP children showed weaker performance in attentional control, cognitive flexibility, and goal setting than FT peers [40]. Additionally, individual studies found that MLP children scored lower in goal setting and backward digit span tasks but not in attentional control [41].

Most studies on reading abilities in preterm children were also conducted with very preterm or extremely preterm children. The results indicate that these children obtain significantly lower results than full-term children in decoding abilities [42,43,44,45,46,47], reading comprehension [48], or in both decoding abilities and reading comprehension [49,50,51,52] at school age, with similar conclusions reached across multiple meta-analyses [53,54,55,56]. However, this body of research may not apply to healthier or moderately preterm populations. In fact, some studies have shown that low-risk PT children perform comparably to FT peers in text comprehension, word reading, and pseudoword decoding [7,57,58,59]. Other work suggests that strong performance in visuospatial memory, working memory, and processing speed may help some PT children compensate for early risks [59,60].

Recent studies involving low-risk PT children have found more mixed results. For instance, ref. [57] concluded that, contrary to predictions, preterm birth did not significantly increase the likelihood of being placed in a poor reading group. Other studies comparing reading skills across gestational age (GA) groups in healthy preterm children report comparable performance to FT children in word reading, pseudoword reading, and reading comprehension at school age [7,58,59,60]. Along the same lines, ref. [58] reported no significant differences in reading abilities among preterm groups with extremely low, very low, and low birthweights between the ages of 8 and 13. The authors of [59] found that very-low-birthweight PT children performed similarly to a group of good readers in reading and spelling, with both groups outperforming a group of children with dyslexia. The authors attributed the strong performance of PT children to strengths in visuospatial memory, working memory, and processing speed.

Far fewer studies have examined how executive functions influence reading development in PT children. Evidence suggests that EFs may play a particularly important role in this population. For example, ref. [57] showed that better reading performance was associated with stronger executive and neuropsychological functioning, such as attention, planning, and cognitive flexibility, regardless of prematurity status. Similarly, ref. [61] found negative effects of prematurity on reading fluency, and these effects were mediated by processing speed and other executive functions, working memory in particular. Other studies have highlighted the role of language and short-term verbal memory in predicting literacy outcomes in very preterm children [62,63].

Findings showing that EF components may show predictive effects on reading, primarily among preterm children, are particularly relevant. The authors of [64] found that phonological awareness and language abilities at 6 years of age predicted reading outcomes at 8 years in PT and FT children, but executive functions were predictive of reading only for PT children. The authors of [7] observed that working memory and phonological awareness predicted reading comprehension in both PT and FT children, but that executive functions were significant predictors of reading only in the PT group. The authors of [65] further emphasized the role of verbal processing speed and visuospatial working memory in reading outcomes among extremely preterm children. However, the contribution of specific EF domains, such as attention or inhibition, remains inconsistent across studies, likely due to variations in age, EF measurement, and reading outcomes assessed.

In sum, different EFs were observed to have direct or indirect effects on reading skills in PT children, although the EFs in question varied, and, in some cases, there were inconsistencies between studies, such as in the case of attention. Yet, more longitudinal research focused on low-risk preterm children using robust EF assessments is needed to clarify these relationships and inform early intervention strategies.

The present study addresses gaps in the literature by examining the effects of executive functions on reading performance in low-risk preterm children across a range of gestational ages. Specifically, it aims to (1) compare the performance of three groups of low-risk preterm children with different GAs and one group of FT children in a series of EFs using cognitive and behavioral scale measures taken at different ages (4, 5, and 8 years); (2) compare the performance of these four GA groups in a reading test that explores decoding skills and comprehension skills administered at 9 years of age; and (3) identify the EFs administered at different times that have an effect on different reading abilities assessed at 9 years of age. With this purpose, a multiple linear regression analysis was performed.

## 2. Materials and Methods

### 2.1. Participants

The original sample consisted of 151 premature children and 49 full-term children and formed part of a longitudinal study. The children were recruited from four hospitals in Galicia (Spain) at birth. The mean GA of the PT children was 32.60 (SD = 2.43; range: 26–36), and that of the FT children was 39.89 (SD = 1.44; range: 37–42). The mean Apgar score of the PT children was 7.87 (SD = 1.43), and that of the FT children was 8.08 (SD = 1.25). No significant difference existed in Apgar scores between both groups (t (197) = −0.909; *p* > 0.05). The group of preterm children was composed of 79 boys and 72 girls, while in the FT group, there were 25 boys and 24 girls. Therefore, there were no significant differences regarding gender between the groups (χ^2^ = 0.025; *p* = 0.874). The PT and FT groups were also similar in terms of maternal education level. Divided into three categories (basic education, high school or technical education, and university education), the PT group included 22 mothers with basic education, 75 with high school or technical education, and 53 with university education, while in the FT group, the frequencies were 13, 19, and 17, respectively. The chi-square test indicated that there was no significant difference between the two groups (χ^2^ = 3.945; *p* = 0.139).

When forming the group of PT children, we excluded those who suffered from cerebral palsy, periventricular leukomalacia (PVL), intraventricular hemorrhage (IVH) greater than grade II, hydrocephalus, genetic malformations, encephalopathy, chromosomal and metabolic syndromes associated with intellectual disability, important motor or sensory (vision or hearing) impairments, or Apgar scores below 6 at 5 min. For these reasons, the children in the PT group could be considered low-risk children.

The data were obtained when the children were between 4 and 9 years of age. In the present study, 145 children participated at 4 years of age: 111 preterm and 34 full-term. At 5 years of age, 140 children participated: 107 preterm and 33 full-term. At 8 years of age, there were 114 children: 88 preterm and 26 full-term. At 9 years of age, the sample consisted of 90 children: 70 preterm and 20 full-term.

**Figure 1:**
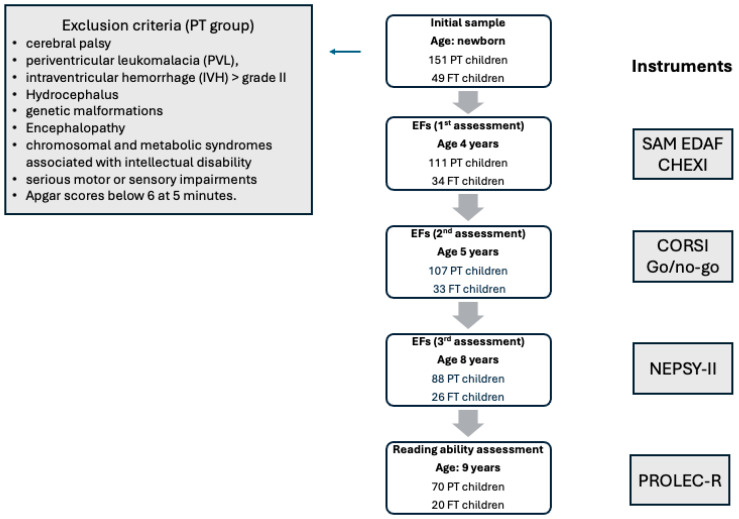
Flow diagram.

The flow diagram shown in Figure 1 provides a graphical representation of the numerical evolution of the sample over time, the exclusion criteria, and the instruments used at each assessment point.

The characteristics of the sample remained consistent over time. At the final assessment point, when participants were 9 years old, the mean gestational age (GA) for the preterm (PT) group was 32.68 weeks (SD = 2.51; range: 27–36), while the full-term (FT) group had a mean GA of 39.75 weeks (SD = 1.65; range: 37–42). Regarding the Apgar scores of the two groups, the values were also very similar to those recorded at the beginning of this study. The preterm children had a mean Apgar score of 7.87 (SD = 1.29), while the full-term children had a mean score of 8.25 (SD = 1.51). Regarding gender, the PT group included 39 boys and 30 girls, while the FT group included 12 boys and 8 girls. There were no significant differences between the groups in gender composition (χ^2^ = 0.077; *p* = 0.782) or in maternal education level (χ^2^ = 2.337; *p* = 0.311). The reduction in the original sample size was due to participant dropout, which was likely increased by the major economic crisis of 2008 and the years that followed. As a result, several families were forced to relocate from their place of residence.

### 2.2. Instruments

A combination of behavioral rating scales and cognitive measures was used to assess EFs at different times. For each assessment, we employed the instruments that were most adequate for that age to assess the EFs of interest. All the tests used in this study have good psychometric values and are internationally used and/or were constructed according to the same parameters as other tests to assess English-speaking children.

The verbal working memory task (*memoria secuencial auditiva: sequential auditive memory (SAM)*) is part of the EDAF (*Evaluación de la Discriminación Auditiva y Fonológica*) [66]. The child is asked to repeat a sequence of words, which progressively increases in number. The total number of correct tries was computed.

The Spanish version of the Childhood Executive Functioning Inventory (CHEXI) [67] was applied to children’s parents to assess working memory and inhibitory control in the children’s daily life. The CHEXI can be used to assess children between 4 and 12 years of age. The CHEXI can be completed by children’s parents or by their teachers, and it includes 24 items, with a 5-point Likert-type format (1 = absolutely uncertain; 5 = very true). Parents rate how much each assertion is a true description of their children’s behavior (e.g., “*Cuando se le pide que haga varias cosas, sólo recuerda la primera o la última*” (when the child is asked to do several things, he/she only remembers the first or the last)). Higher scores indicate greater difficulty in working memory and inhibitory control, and lower scores indicate fewer difficulties in working memory and inhibitory control. The total scores in working memory and inhibitory control were used in this study.

The CORSI ordering task [68,69] was used to assess non-verbal sequential working memory. Colored blocks from an array of 9 blocks are highlighted in a certain sequence. The children must repeat the sequence immediately. Each sequence has a certain length, increasing from 2 to a maximum of 9 blocks. The task is finished when the child fails two trials of a given length. The PEBL implementation [70] was used. A portable computer with a touch screen was used (an HP Pavilion Sleekbook TouchSmart 15-b153sg, Hewlett Packard European Regulatory, Boeblingen, Germany). The total CORSI score, which provides the PEBL implementation (the number of correct answers multiplied by the block span), was used for the analysis.

The Go/No-Go task [71,72] was used to evaluate both sustained attention and inhibitory control. It consisted of two distinct phases, each including 50 trials. During the first phase, children were instructed to press a key whenever a designated target stimulus (the letter “R”) appeared (the Go stimulus) and to withhold their response when a different letter (“P”) was shown (the No-go stimulus). In the second phase, the stimuli were reversed: participants had to respond to the letter “P” and inhibit their response to the letter “R”. The task consisted of 100 trials in total, with a target-to-non-target ratio of 80:20. Each letter was displayed for 500 milliseconds, followed by a 1500-millisecond interval before the next stimulus. Before beginning each phase, the children completed a brief practice session to ensure their understanding of the task. The task was administered using the PEBL software [70] on an HP Pavilion Sleekbook TouchSmart 15-b153sg with a touchscreen interface. Two main performance measures were recorded: the number of correct responses (an index of sustained attention) and the reaction time for incorrect responses (an indicator of impulsivity) [71,73].

In addition, the Spanish version of the NEPSY-II attention and executive function domain [74] was administered to assess cognitive functioning in 8-year-old participants. The NEPSY-II battery is designed to evaluate neuropsychological development in children aged 3 to 16 years. For this study, only tasks within the attention and executive functioning domain relevant to this age group were included: auditory attention and cognitive flexibility, animal classification, inhibition, and clocks. The design fluency task was excluded due to the high level of difficulty it posed for the children.

The auditory attention and response set subtests assessed selective auditory attention and the ability to sustain it over time, as well as cognitive flexibility, specifically, the ability to shift between response rules and inhibit previously learned patterns. The animal classification task measured concept formation and the capacity to alternate between different conceptual categories. The inhibition subtest evaluated the child’s ability to suppress automatic responses and switch between response types. Finally, the clocks task assessed a combination of planning, organization, visual–spatial, and visual–perceptual skills, along with time-telling abilities using analog clocks.

The NEPSY-II provides several types of scores: primary scale scores, process scores, and contrast scores. In this study, combined scores, those derived from integrating multiple aspects of each task, were used. For example, the inhibition naming combined score includes both task completion time and error rate. The following NEPSY-II scores were analyzed: the combined scores for auditory attention and cognitive flexibility, animal classification, and the total inhibition score (a composite of naming, inhibition, and switching components, IND, INI, and INC in the Spanish version), as well as the score for the clocks task.

The *Batería de evaluación de los procesos lectores, revisada* (PROLEC-R) was administered to the participants when they were 9 years old (±1 month) to assess reading capacity [75]. This test is used with children between 6 and 12 years of age. The PROLEC-R consists of nine tasks. The first eight tasks were considered in the present research, since the last one (listening) is used for clinical purposes to identify discrepancies between written and oral comprehension.

The first task—the identification of letter (letter names)—consists of a list of 20 letters (e.g., “g”). In this task, the researcher points to the letter, and the child reads it out loud. The same–different task consists of 20 pairs of words. Ten of the pairs are the same words. In the other ten, one of the words is a real noun, and the other one is a pseudoword (e.g., marguen–margen). The child is asked to look carefully at these pairs and point to those that are the same. The word-reading task consists of a list of 40 words that vary in length, frequency of use, and the complexity of their syllabic structure (e.g., “peine” (comb)). The pseudoword-reading task consists of a list of 40 invented words (e.g., “gloro”). In each task, the child receives a precision score, measured as the sum of the correct answers, and a speed score, measured as the time taken to complete the task. A combined score (efficiency) is calculated by dividing the precision score by the speed score and multiplying the result by 100. The efficiency score was used for the analyses in this study.

In the grammatical structures task, the child is presented with a sentence and four pictures depicting different events. The child is asked to read the sentence out loud and then point to the picture that correctly depicts the event. Overall, there are 16 items, and only accuracy is considered. In the punctuation marks test, the child is asked to read a text in which there are different punctuation marks, such as periods, commas, question marks, and exclamation points. In this test, both accuracy and time are measured.

The sentence comprehension task consists of three parts. First, the child is asked to read three sentences and do exactly what they indicate (e.g., put the pencil on the notebook). Then, the child is given another six sentences and a sheet of paper where they can draw whatever is required. Finally, there are seven sentences presented together with pictures (e.g., the sentence is “the horse is smaller than the elephant”, where one pair of pictures shows the elephant bigger than the horse, the same size of both animals, and the pair depicting the sentence). The child is asked to read the sentences and indicate the picture depicting the sentence.

The text comprehension task consists of two narrative and two expository texts. For each text, the children are asked to respond to 4 written questions, with 16 total responses (e.g., “¿*Para qué sacó varias monedas de la hucha*?” (Why did he take a few coins out of the piggybank?)). The child receives one point for each correct answer given. In all cases, raw scores were used for the analyses, not percentile or scalar scores.

The PROLEC-R provides valuable insights for educational planning and early detection of reading difficulties such as dyslexia, offering a comprehensive profile of the child’s reading development.

### 2.3. Procedure

Parents’ consent and the authorization of the Galician Ethics Committee of Clinical Research (2008/010) were obtained prior to the participation of the children in this study. Data were collected by trained researchers who visited the children’s homes on four occasions within a 6-year interval.

The CHEXI was given to the parents for them to fill in, and the verbal working memory task of the EDAF was administered to the children when they were 4 years of age (±1 month). When the children were 5 years old (±1 month), the CORSI test and the Go/No-Go task were applied. The NEPSY-II was administered when the children were 8 years old (+1 month). Finally, the PROLEC-R was administered to the children when they were 9 years old (±1 month).

### 2.4. Analyses Performed

One-factor ANOVA was carried out to compare the performance of preterm and full-term children in reading skills and executive functions. For the mean comparisons (ANOVA), the participants were divided into 4 groups according to gestational age: (1) extremely premature and very premature children with a GA below 32 weeks of gestation, (2) moderately preterm children with a GA of 32 or 33 weeks, (3) late preterm children with a GA between 34 and 36 weeks, and (4) full-term children with a GA greater than or equal to 37 weeks. The number of participants in each gestational age group varied depending on the age of assessment and the test, and their frequency is indicated in the ANOVA results table. When the homogeneity of variances was not confirmed by the Levene test, the Brown–Forsythe test was used. In the case of significant differences between groups, the Bonferroni post hoc test was applied to identify the existing differences between gestational age groups.

We conducted Kolmogórov–Smirnov tests to determine whether the data followed a normal distribution. In many cases, the normality assumption was not met. In those cases, we used the non-parametric Kruskal–Wallis test.

Pearson’s bilateral correlation of EFs and reading variables were also obtained. IBM SPSS 29 was used to perform the ANOVA, Kruskal–Wallis, and Pearson’s correlation analyses.

Six multiple linear regression analyses were conducted with the assistance of IBM SPSS Amos 28 Graphics. To make the multiple regression analyses more powerful, all the children were put together, given that there were no significant differences between them. The aim was to estimate the effects of the EDAF sequential auditory memory score, CHEXI working memory total score, CHEXI inhibitory control total score, CORSI total score, correct responses to Go/No-Go, reaction time score on Go/No-Go errors, NEPSY auditory attention combined scale score, NEPSY cognitive flexibility combined scale score, NEPSY animal classification combined scale score, NEPSY clocks scale score, and NEPSY inhibition total score in each of the following dependent variables: (1) letter names, (2) same–different, (3) word reading, (4) pseudoword reading, (5) grammatical structures, (6) punctuation marks, (7) sentence comprehension, and (8) text comprehension.

## 3. Results

### 3.1. EF Comparisons

Table 1 provides a summary of the ANOVA results examining differences in executive function scores between the gestational age groups.

Table 1 shows that the only statistically significant difference identified by the ANOVA was in the CHEXI inhibitory control measure. Post hoc comparisons using the Bonferroni test revealed that this effect was due to a significant difference between the 32–33 and 34–36 gestational week groups. Specifically, children in the 34–36 week group scored higher on this measure, suggesting greater difficulties with inhibitory control compared with the 32–33 week group.

### 3.2. Reading Skills Comparisons

Table 2 presents the ANOVA results comparing PROLEC-R performance across the gestational age groups. No statistically significant differences were observed in any of the assessed components, including letter identification, same–different discrimination, word and pseudoword reading, understanding of grammatical structures and punctuation, and sentence and text comprehension.

When the assumption of normality was not met for certain executive function or reading ability variables, the Kruskal–Wallis test was applied. As the results were consistent with those obtained from the ANOVA—showing no significant differences between groups—we chose to report the ANOVA results, as they provide more detailed information. Nonetheless, the results of the Kruskal–Wallis test are provided in Appendix A.1.

### 3.3. Effects of EFs on Reading Skills

As practically no significant differences were found in the EFs among the groups, and no difference was found in the reading test (PROLEC-R), all the children were put together for the multiple regression analysis.

The results of the multiple linear regression analysis are presented in Table 3 for the combined scores (efficiency score) of five tasks (letter names, same–different, word reading, pseudoword reading, and punctuation marks) and the total score of the remaining three (grammatical structures, sentence comprehension, and text comprehension) (see Table 3). The variance explained by EFs for the different reading measures reached modest-to-moderate effects.

For the dependent variable *letter names*, the variance explained by the EFs reached 0.29 (R^2^). CHEXI working memory (*p* < 0.001) and CORSI total score (*p* = 0.044) were the independent variables that showed a significant effect.

*Same–different* variance was explained up to 0.15 (R^2^) by the model, and sequential auditory memory (EDAF) (*p* = 0.004) was the only independent variable that had a significant effect on this variable.

The variance explained for *word reading* reached 0.18 (R^2^), and, again, sequential auditory memory (EDAF) was the only variable that showed a significant effect (*p* < 0.001).

The variance explained for *pseudoword reading* reached 0.11, and no single independent variable showed a significant effect on it.

The variance explained for *grammatical structures* was 0.17 (R^2^), and cognitive flexibility was the only variable that showed a significant effect on it (*p* = 0.021).

For *punctuation marks*, the variance explained reached 0.08 (R^2^), and no single independent variable had a significant effect.

*Sentence comprehension* was explained by the model up to 0.30 (R^2^), and four EFs showed a significant effect on this variable: CHEXI inhibitory control (*p* = 0.32), correct responses Go/No-Go (*p* < 0.001), NEPSY-II cognitive flexibility (*p* = 0.026), and NEPSY-II animal classification (*p* = 0.033).

Finally, *text comprehension* was explained by the model up to 0.21 (R^2^), and two EFs showed a significant effect on it: CORSI total score (*p* = 0.007) and NEPSY-II clocks (*p* = 0.012).

In addition, we checked the results of the model for punctuation mark speed and precision, just to see if any effect could be observed with these measures. *Punctuation mark speed* was explained up to 0.14 (R^2^) by the model, and CHEXI working memory showed a significant effect on it (standardized beta = 0.262; *p*= 0.18). In the case of *punctuation mark precision*, the variance explained (R^2^) reached 0.12, and the only independent variance that had a significant effect was the NEPSY clocks scale score (standardized beta = 0.255, *p* = 0.013).

In the same way, we also checked the results of the model for pseudoword-reading speed and precision. In the case of speed, the variance explained reached 0.14, and no single variance had a significant effect. For pseudoword reading, however, the variance explained (R^2^) reached 0.35, and several variables had significant effects: CHEXI working memory (beta = 0.210; *p* = 0.015), CHEXI inhibitory control (beta = −0.312; *p* < 0.001), CORSI total score (beta = 0.180; *p* = 0.043), NEPSI-II auditory attention (−0.280; *p* = 0.001), NEPSY-II cognitive flexibility (beta = 0.191; *p* = 0.029), and NEPSY-II clocks (beta = 0.244; *p* = 0.012).

The results of Pearson’s correlation are offered in Appendix A.2.

## 4. Discussion

The first and second objectives of this study were to compare the performance of the four groups of children with different gestational ages in different EF measures taken at 4, 5, and 8 years of age, and in reading skills measured at 9 years of age. The third objective of this study was to identify the EFs that have effects on different reading abilities.

The ANOVA results indicate that, overall, no significant differences were observed between the three preterm groups with different gestational ages and the full-term group in nearly all executive function measures. The only exception was found in the CHEXI inhibitory control scores. A post hoc Bonferroni test revealed that this difference was due to a significant difference between the moderately and late preterm groups, with the moderately preterm children exhibiting lower inhibitory control. Importantly, no significant differences were identified between the full-term group and any of the preterm groups across a wide range of executive function measures, including sequential auditory memory (SAM), working memory and inhibitory control (CHEXI), non-verbal sequential memory (CORSI), sustained attention, impulsivity, and inhibition (the Go/No-Go task), and auditory attention, cognitive flexibility, animal classification, inhibition, and planning/organization (NEPSY-II). The mean scores obtained by full-term participants were consistent with normative expectations based on the Spanish standardizations of the CHEXI [67], NEPSY-II [74], and the verbal working memory task (EDAF) [66].

Thus, the results from our sample of low-risk preterm children do not support previous research suggesting that preterm children generally show lower executive function performance compared with their full-term peers [27,28,30,31,32,33,34,35,36,38,42]. Notably, most of those studies focused on very or extremely preterm populations, with the exception of [40,41], who examined moderate and late preterm children. However, in [40], group differences tended to decrease with increasing gestational age, and the outcomes of moderate preterm children were not specifically compared with those of late preterm children, which represents a limitation of this study. Additionally, another limitation of this study [40] was the lack of control for potential confounding factors (e.g., genetic, biomedical, or socio-demographic variables). In [41], although preterm children with congenital abnormalities and genetic syndromes were excluded, other confounding biomedical factors may still have been present. In contrast, our findings are consistent with studies that reported no significant differences in executive functioning between low-risk preterm and full-term children [7,38,39]. Importantly, our conclusions are supported by both behavioral rating scales and direct cognitive assessments administered across multiple developmental stages. The absence of significant differences at ages 4, 5, and 8 suggests that the developmental trajectory of executive functions was comparable between low-risk preterm and full-term children.

In relation to the second objective, the results indicate that no significant difference was found between the gestational age groups in any of the measures of reading ability (PROLEC-R). Importantly, there was no significant difference between the FT group and any of the PT groups with different GAs. The FT children obtained standard scores in the different measures of the PROLEC-R: letter names, same–different, word reading, pseudoword reading, grammatical structures, punctuation marks, sentence comprehension, and text comprehension, which indicates that the FT children did not have reading difficulties [76].

The fact that the PT children could be considered as low-risk may be responsible for the results found, and this could greatly differ from the results obtained by other studies carried out on, in general terms, very or extremely preterm children [27,42,44,45,46,47,48,49,50,51,52,54,55,56]. The results we found indicate that reading skills seem to develop in low-risk PT children in a way similar to those of FT children, coinciding with other studies [7,57,59,60].

In relation to the third objective of this study, the results show a different effect of different executive functions depending on the complexity of the reading process. In the case of lower-level processes (letter identification, same–different, pseudoword reading, and word reading), which deal with spelling processes, those executive functions that have a significant effect on them are also related to lower-level executive functions, such as working memory. This is the case with working memory (CHEXI), which very significantly contributes to the explanation of letter names. Non-verbal sequential memory (the CORSI total score) also contributes to the explanation of letter names. Sequential auditory memory (verbal memory) (EDAF) highly contributes to the explanation of the results obtained in same–different and word reading. Working memory and non-verbal sequential memory are particularly related to the identification of letters and their names. In contrast, those memory processes more related to auditory (verbal) memory contribute to the explanation of same–different and word reading. In the case of same–different, the children were asked to indicate if the pairs of words were the same or if they were different. In same–different, as well as in word reading, the recognition of the word plays an important role. Therefore, it is not strange for verbal–auditory memory to play an important role in these two tasks. The relevance of verbal and non-verbal working memory has been pointed out by several studies [7,10,22,61,63,65], although we identified differences in their effects.

In contrast, those tasks that are of a higher complexity and are related to complex meaning formation, such as grammatical structures, sentence comprehension, and text comprehension, are more related to complex executive functions. Cognitive flexibility (NEPSY-II) is involved in the explanation of grammatical structures and sentence comprehension, coinciding with the results found by other authors [10,23,57]. Planning, goal setting, and structural organization (NEPSY-II clocks) contribute to the explanation of the variance of text comprehension. Other authors [10,21,22,57] also found an effect of planning on reading comprehension. Finally, conceptual capacity and flexibility (NEPSY-II animal classification) also contribute to the explanation of the results in sentence comprehension. In this last case, the sign of the effect is negative, although we think this is a measurement error since the correlation between cognitive flexibility and animal classification is very high (r = 0.395; *p* < 0.01).

Inhibitory control also shows effects on sentence comprehension (CHEXI inhibitory control, and, to a higher extent, on the Go/No-Go task), indicating that the capacity of inhibiting first options and controlling impulsivity helps in the understanding of sentences. Other authors have also highlighted the importance of the capacity to suppress irrelevant information in reading comprehension [19,20,23].

In contrast with what occurs in lower-level reading processes, working memory does not show effects on higher-level reading processes, such as grammatical structures, sentence comprehension, or text comprehension, although there could be an indirect or mediated effect (possibly through language) on text comprehension, as [26] have found. Even having good non-verbal sequential memory (CORSI) does not help text comprehension performance, all the contrary.

Therefore, there seems to be a differential effect of executive functions depending on the reading process under scrutiny [14].

Although no significant effect of any EF was observed on punctuation marks and pseudoword reading when combined measures (efficiency scores) were taken into consideration, the landscape changed when speed or precision were considered. CHEXI working memory had a negative effect (remember that negative scores indicate good memory or good inhibitory control in CHEXI) on punctuation marks when speed was considered, which indicates that good memory does not help to have high scores in this task. More interesting was the result found when the accuracy or precision score was considered. In this case, good accuracy in punctuation marks was explained, in part, by the good capacity of planning and structural ordering (NEPSY-II clocks), indicating that the correct reading of punctuation marks is related to a global perception and goal perception of the sentence.

In relation to pseudoword reading, when speed was considered as a dependent variable, no executive function had a significant effect, and the variance explained was low (0.10). The situation dramatically changed when pseudoword-reading accuracy was considered as a dependent variable. This time, the variance explained reached 0.35 (R^2^), and several variables had significant effects. Firstly, CHEXI inhibitory control showed the highest impact, indicating that this EF had a relevant role in pseudoword reading, by inhibiting first impression reactions (probably a similar real word) and controlling impulsivity. Cognitive flexibility (NEPSY-II) also had an influence. Children with higher flexibility tend to read pseudowords with better precision than children with lower cognitive flexibility, since they are open to other options that are not a known word. Probably, both inhibitory control and cognitive flexibility work together. Everyday working memory (CHEXI) has a negative impact, indicating that working memory does not help pseudoword-reading accuracy. In contrast, non-verbal sequential memory (CORSI) has a low, although significant, effect. Finally, auditory attention (NEPSY-II), as well as planning, goal setting, and structural organization (NEPSY-II clocks), have negative effects, indicating that these executive functions could even have a detrimental effect on pseudoword-reading accuracy, indicating the arbitrary and non-familiar character of pseudowords.

Long-lasting effects of executive functions on different reading abilities can be observed. Measures taken at 4 and 5 years of age still show a clear effect on reading skills at 9 years. These results agree with the findings obtained by other scholars with preterm children [47,57,64].

### Study Limitations

Over this 9-year longitudinal study, there was a significant loss of participants. While participant attrition is a common feature of longitudinal studies, in our case, it was exacerbated by the length of this study and by the fact that it began during the major economic crisis of 2008, which forced some families to relocate. Undoubtedly, the reduced number of participants by the end of this study—when reading skills were assessed—means that the results should be interpreted with caution. This circumstance also reduced the statistical power of the tests used.

This study focused on specific variables, namely, certain executive functions and reading ability. It is clear that other variables not considered here, particularly those related to language development, may have influenced the results. It is also possible that indirect effects of executive functions on reading ability could have been observed through these unmeasured mediating variables. This represents another limitation of this study, which was primarily aimed at highlighting the influence of executive functions on reading skills.

Further research extending the study of low-risk preterm children into adolescence and adulthood would also be desirable in order to better understand the long-term effects of prematurity.

## 5. Conclusions

Unlike extremely and very PT children, low-risk PT children of different GAs do not appear to perform worse than FT children in either executive functions or reading skills, at least in the executive functions that were assessed in the present study and the reading skills measured. The robustness of the findings we obtained with EFs leans on the fact that they were obtained through the administration of behavioral rating scales and cognitive measures throughout different ages.

The effects of EFs on reading skills are low to moderate. The effects of the different EFs vary depending on the reading process under observation. Verbal and non-verbal working memory had a positive significant effect on decoding skills (letter names, same–different, and word reading). Higher-order EFs (cognitive flexibility and planning), as well as inhibitory control, showed positive effects on reading comprehension skills (grammatical structures, punctuation marks accuracy, sentence comprehension, and text comprehension).

There are important effects of EFs measured at early ages (4 and 5 years) on reading skills measured at 9 years of age.

## Figures and Tables

**Table 1:** ANOVA results for executive functions.

Variable	Group	N	Mean	SD	df	F	η^2^	*p*
CHEXI working memory total score	<32	36	30.19	10.40				
	32–33	32	27.00	8.21				
	34–36	43	30.58	11.07				
	>37	34	30.11	7.43				
	Total	145	29.58	9.55	3.141	1.02	0.02	0.38
CHEXI inhibitory control total score	<32	36	31.83	9.37				
	32–33	32	30.31	7.32				
	34–36	43	35.13	6.87				
	>37	34	34.08	6.30				
	Total	145	33.00	7.70	3.141	3.02	0.06	0.03
EDAF sequential auditory memory score	<32	34	5.41	2.36				
	32–33	32	5.28	2.78				
	34–36	42	5.98	2.51				
	>37	34	5.79	2.54				
	Total	142	5.64	2.54	3.138	0.58	0.01	0.62
CORSI total score	<32	32	10.50	7.42				
	32–33	28	9.25	8.30				
	34–36	37	11.78	8.47				
	>37	31	10.64	7.60				
	Total	128	10.63	7.93	3.125	0.54	0.013	0.65
Correct responses Go/No-Go total score	<32	30	73.10	11.33				
	32–33	26	68.80	12.16				
	34–36	40	73.52	10.54				
	>37	33	70.39	18.17				
	Total	129	71.67	13.34	3.125	0.86	0.02	0.45
Reaction time Go/No-Go errors	<32	29	606.15	188.66				
	32–33	25	557.90	92.02				
	34–36	40	636.30	171.34				
	>37	32	609.51	196.09				
	Total	126	607.00	170.45	3.104	1.14 *	0.026	0.33
NEPSY auditory attention combined scale score	<32	27	12.55	2.60				
	32–33	27	13.07	1.93				
	34–36	33	12.90	2.11				
	>37	26	12.88	2.02				
	Total	113	12.85	2.16	3.109	0.26	0.007	0.84
NEPSY cognitive flexibility combined scale score	<32	27	12.33	3.43				
	32–33	27	12.70	3.16				
	34–36	33	13.15	3.29				
	>37	25	14.48	2.67				
	Total	112	13.14	3.22	3.105	2.27 *	0.59	0.85
NEPSY animal classification combined scale score	<32	27	8.51	1.36				
	32–33	24	9.41	2.24				
	34–36	32	8.28	1.54				
	>37	26	9.00	1.32				
	Total	109	8.76	1.67	3.105	2.56	0.68	0.58
NEPSY inhibition total score	<32	27	32.62	1.37				
	32–33	26	34.11	1.10				
	34–36	33	33.03	0.94				
	>37	26	34.96	1.09				
	Total	112	33.63	0.56	3.108	0.85	0.23	0.46
NEPSY clocks scale score	<32	28	6.21	4.12				
	32–33	26	6.61	4.07				
	34–36	34	6.88	4.54				
	>37	26	5.76	4.39				
	Total	114	6.40	4.26	3.110	0.36	0.01	0.77

* Brown–Forsythe test.

**Table 2:** ANOVA results for PROLEC-R.

Variable	Group	N	Mean	SD	df	F	η^2^	*p*
Letter names	<32	18	160.80	27.49				
	32–33	23	156.54	40.31				
	34–36	28	158.83	38.42				
	>37	20	168.48	30.17				
	Total	89	160.80	34.94	3.85	0.45	0.01	0.71
Same–different	<32	18	32.59	6.95				
	32–33	22	35.05	8.34				
	34–36	27	36.26	8.88				
	>37	20	37.91	5.73				
	Total	87	35.57	7.81	3.83	1.60	0.05	0.19
Word reading	<32	18	95.08	38.22				
	32–33	23	89.65	19.89				
	34–36	28	90.24	31.61				
	>37	20	90.94	28.30				
	Total	89	91.22	29.40	3.85	0.13	0.00	0.94
Pseudoword reading	<32	18	61.36	18.81				
	32–33	23	56.23	13.17				
	34–36	28	58.15	16.36				
	>37	19	97.99	161.15				
	Total	88	66.91	76.42	3.84	1.13 *	0.04	0.36
Grammatical structures	<32	18	14.28	1.67				
	32–33	22	13.82	2.26				
	34–36	28	14.61	1.61				
	>37	20	14.70	1.52				
	Total	88	14.36	1.79	3.84	1.10	0.03	0.35
Punctuation marks	<32	14	23.26	3.57				
	32–33	20	22.08	5.03				
	34–36	22	23.35	5.37				
	>37	14	26.41	19.02				
	Total	70	23.58	9.41	3.66	0.59	0.02	0.62
Sentence comprehension	<32	18	15.72	0.57				
	32–33	23	15.48	1.12				
	34–36	28	15.75	0.585				
	>37	19	15.63	0.59				
	Total	88	15.65	0.75	3.84	0.60	0.02	0.61
Text comprehension	<32	18	14.39	1.19				
	32–33	23	14.57	1.19				
	34–36	28	14.46	1.07				
	>37	19	14.32	1.20				
	Total	88	14.44	1.14	3.84	0.178	0.00	0.91

* Brown–Forsythe test.

**Table 3:** Multiple linear regression analysis: beta-standardized regression weights, significance levels, and efficiency scores.

	IV	Letter Names	Same–Different	Word Reading	Pseudoword Reading	Grammatical Structures	Punctuation Marks	Sentence Comprehension	Text Comprehension
DV									
CHEXI working memory		**−0.426** **(<0.001)**	−0.091 (0.357)	0.041 (0.668)	0.153 (0.129)	0.137 (0.161)	−0.078 (0.501)	−0.060 (0.507)	−0.167 (0.080)
CHEXI inhibitory control		0.087 (0.329)	0.099 (0.317)	−0.135 (0.162)	−0.095 (0.350)	−0.051 (0.602)	−0.016 (0.888)	**0.193** **(0.032)**	0.022 (0.814)
Sequential auditory memory EDAF		0.115 (0.200)	**0.291** **(0.004)**	**0.334** **(<0.001)**	−0.075 (0.467)	0.132 (0.180)	0.001 (0.993)	0.029 (0.753)	0.004 (0.964)
CORSI total score		**0.186** **(0.044)**	−0.053 (0.605)	−0.109 (0.276)	0.078 (0.456)	0.123 (0.224)	−0.116 (0.330)	−0.167 (0.074)	**−0.264** **(0.007)**
Correct responses Go/No-Go		−0.141 (0.128)	0.046 (0.658)	0.147 (0.141)	−0.061 (0.558)	0.153 (0.131)	0.123 (0.307)	**0.357** **(<0.001)**	0.131 (0.185)
Reaction time Go/No-Go errors		−0.069 (0.462)	−0.006 (0.956)	−0.002 (0.980)	0.104 (0.322)	0.019 (0.849)	0.066 (0.586)	0.035 (0.707)	−0.019 (0.852)
NEPSY auditory attention combined scale		0.152 (0.097)	0.080 (0.430)	−0.011 (0.915)	−0.025 (0.808)	−0.001 (0.996)	−0.109 (0.360)	−0.056 (0.546)	−0.128 (0.190)
NEPSY cognitive flexibility combined scale		0.040 (0.661)	0.154 (0.128)	0.066 (0.505)	0.149 (0.148)	**0.230** **(0.021)**	0.122 (0.302)	**0.205** **(0.026)**	0.006 (0.949)
NEPSY animal classification combined scale		0.080 (0.381)	0.013 (0.901)	−0.434 (0.802)	0.033 (0.751)	−0.159 (0.111)	0.071 (0.552)	**−0.196** **(0.033)**	0.018 (0.855)
NEPSY inhibition total score		0.014 (0.878)	0.105 (0.305)	−0.004 (0.966)	0.045 (0.669)	0.110 (0.276)	0.050 (0.677)	0.091 (0.330)	0.151 (0.125)
NEPSY clocks scale score		−0.025 (0.784)	0.016 (0.873)	0.103 (0.300)	−0.152 (0.142)	−0.002 (0.988)	−0.051 (0.668)	0.083 (0.370)	**0.244** **(0.012)**
R^2^		0.29	0.15	0.18	0.11	0.17	0.08	0.30	0.21

## Data Availability

The data presented in this study are available on request from the corresponding author due to privacy reasons.

## Abbreviations

The following abbreviations were used in this manuscript:

CHEXI	The Childhood Executive Functioning Inventory
EDAF	*Evaluación de la Discriminación Auditiva y Fonológica*
EFs	Executive functions
EPT	Extremely preterm
FT	Full-term
GA	Gestational age
IVH	Intraventricular hemorrhage
MLP	Moderate and late preterm
NEPSY-II	Evaluación Neuropsicológica Infantil
PEBL	The Psychology Experiment Building Language
PT	Preterm
PVL	Periventricular leukomalacia
RT	Reaction time
SAM	Sequential auditive memory

## Appendix A

### Appendix A.1. Results of Kruskal–Wallis Test

**Table A1:** Results of Kruskal–Wallis Test.

Executive Functions	H	df	Significance
EDAF seq. auditory memory	1.184	3	0.757
CORSI total score	1.933	3	0.586
Reaction time Go/No-Go errors	4.897	3	0.179
NEPSY auditory attention	0.113	3	0.990
NEPSY cognitive flexibility	7.109	3	0.069
NEPSY animal classification	4.881	3	0.181
NEPSY inhibition	3.791	3	0.285
NEPSY clocks	1.205	3	0.752
Reading abilities			
Letter names	0.816	3	0.846
Same–different	5.392	3	0.145
Word reading	1.080	3	0.782
Pseudoword reading	1.873	3	0.599
Grammatical structures	4.251	3	0.236
Punctuation marks	0.627	3	0.890
Sentence comprehension	1.435	3	0.697
Text comprehension	1.109	3	775

### Appendix A.2. Results of Correlation Analysis

**Table A2:** Results of Correlation Analysis.

	1	2	3	4	5	6	7	8	9	10	11	12	13	14	15	16	17	18	19
1 Letter names	1																		
2 Same–different	201	1																	
3 Word reading	0.628 ***	0.137	1																
4 Pseudoword reading	0.537 ***	0.074	0.629 ***	1															
5 Gramma-tical str.	0.081	0.171	−0.011	0.168	1														
6 Punctuation marks	0.073	−0.083	0.191	0.371 **	0.150	1													
7 Sentence compr.	−0.085	0.229	0.022	0.106	0.142	0.118	1												
8 Text compr.	0.047	0.151	0.169	0.068	0.078	0.253	0.411 **	1											
9 EDAF SAM	0.269	0.300 *	0.548 ***	0.303 *	0.284 *	0.068	0.140	0.085	1										
10 CHEXI WM	−0.386 **	−0.095	−0.318 *	−0.192	0.112	−0.122	−0.088	−0.274	−0.291 *	1									
11 CHEXI IC	−0.306 *	0.003	−0.307 *	−0.279	0.021	−0.041	0.013	−0.215	−0.269	0.621 **	1								
12 CORSI total	0.204	0.088	0.078	−0.019	0.244	−0.070	−0.090	0.011	0.140	−0.005	−0.036	1							
13 Total Go	−0.035	−0.030	0.152	0.057	0.223	0.194	0.196	0.247	0.142	−0.199	−0.083	0.196	1						
14 RT Go	−0.076	−0.006	−0.020	−0.009	0.204	0.123	0.162	0.244	0.159	−0.199	0.008	−0.054	0.183	1					
15 NEPSY AA	0.180	0.192	0.061	−0.053	0.116	−0.055	−0.096	−0.070	0.151	−0.036	−0.233	0.191	0.057	−0.052	1				
16 NEPSY CF	0.100	0.281 *	0.031	−0.111	0.204	0.044	0.050	−0.059	0.176	−0.192	−0.105	0.179	−0.020	0.029	0.435 **	1			
17 NEPSY AC	0.062	0.100	−0.004	−0.048	−0.110	0.155	0.050	0.033	−0.086	−0.102	−0.161	0.287	0.166	−0.002	0.367 **	0.395 **	1		
18 NEPSY clocks	0.184	0.231	0.196	0.061	0.043	−0.079	0.002	0.230	0.131	−0.353 *	−0.313*	0.397 **	−0.037	−0.157	0.223	0.334 *	0.217	1	
19 NEPSY inhibition	−0.012	0.068	0.035	−0.090	−0.025	0.038	0.166	0.076	0.084	−0.071	−0.067	−0.015	−0.101	0.011	0.264	0.172	0.025	0.142	1

* <0.05 bilateral. ** <0.01 bilateral. *** <0.001 bilateral. Legend: Letter names = letter names efficiency score. Same–different = same–different efficiency score. Word reading = word-reading efficiency score. Pseudoword reading = pseudoword reading efficiency score. Grammatical str. = grammatical structures. Punctuation marks = punctuation marks efficiency score. Sentence compr. = sentence comprehension. Text compr. = text comprehension. EDAF SAM = EDAF sequential auditory memory score. CHEXI WM = CHEXI working memory total score. CHEXI IC = CHEXI inhibitory control total score. CORSI total = CORSI total score. Correct go = Go/No-Go correct responses total score. RT go = Go/No-Go task reaction time errors score. NEPSY AA = NEPSY-II auditory attention combined scale score. NEPSY CF = NEPSY-II cognitive flexibility combined scale score. NEPSY AC = NEPSY-II animal classification combined scale score. NEPSY clocks = NEPSY-II clocks scalar score. NEPSY inhibition = NEPSY-II inhibition total score.
